# Job Satisfaction, Intention to Leave, and Related Factors among Foreign-Educated Nurses in Japan: A Cross-Sectional Study

**DOI:** 10.1155/2023/9686746

**Published:** 2023-10-31

**Authors:** Jing Hua, Akiko Kondo, Congcong Wang, Sambuu Ganchuluun

**Affiliations:** ^1^International Nursing Development, Graduate School of Health Care Sciences, Tokyo Medical and Dental University, 1-5-45 Yushima, Bunkyo-ku, Tokyo 113-8519, Japan; ^2^Mongolia-Japan Hospital University of Medical Sciences, Ulaanbaatar, Mongolia

## Abstract

**Aims:**

To examine the association between job satisfaction and the intention to leave and explore the factors associated with job satisfaction or the intention to leave among foreign-educated nurses in Japan.

**Design:**

A cross-sectional design was used, and data were collected through an online survey of nurses who were born and received their basic nursing education outside of Japan but are currently working as registered nurses in Japan. Data were analyzed across two phases: the first explored the related factors with intention to leave and job satisfaction using bivariate analysis and regression through IBM SPSS; the second examined the theoretical framework model using a structural equation model through IBM Amos.

**Results:**

Data from 180 participants (effective response rate: 87.4%) were analyzed. Overall, foreign-educated nurses reported moderate job satisfaction level in Japan. The final model showed good fit indices, indicating that higher workplace discrimination, lower Japanese language satisfaction, and not receiving orientation were predictors of foreign-educated nurses' lower job satisfaction. Lower job satisfaction, fewer years of nursing practice in Japan, single status, and higher language satisfaction predicted a higher intention to leave.

**Conclusion:**

This study provides incremental evidence of a negative relationship between job satisfaction and intention to leave among foreign-educated nurses in Japan. Workplace discrimination was the main predictor of nurses' job dissatisfaction and indirectly correlated with their intention to leave, as mediated by job satisfaction. *Implications*. Our study suggests that managers should provide a supportive and equal work environment, including implementing policies to reduce workplace discrimination and providing adequate support programs to enhance foreign-educated nurses' job satisfaction and reduce their turnover intention.

## 1. Introduction

With the global nursing shortage set to increase to over 36 million by 2030 [1], recruiting foreign-educated nurses has become a worldwide phenomenon. Foreign-educated nurses are those who were born and received nursing education in their countries of origin and are now working overseas [2]. More than 30% of nurses in Switzerland, Australia, and Israel are foreign-educated [3]. Japan has a small number of foreign-educated nurses because of public perception that the acceptance of migrants may threaten Japan's hermetic culture [4]. However, a national Japanese study by Hirano et al. [5] indicated that >80% of hospital managers were interested in recruiting foreign-educated nurses. Global trends also indicate an increasing number of foreign-educated nurses in these receiving countries [3].

Foreign-educated nurses who want to work in Japan as registered nurses are required to undertake Japan's national nursing examination to obtain a nursing license [6]. In Japan, there are two main methods of recruiting foreign-educated nurses. First, the Japanese government began to attract healthcare workers, including nurses, nurse assistants, and others from Indonesia (2008), the Philippines (2009), and Vietnam (2014) under the Economic Partnership Agreement (EPA). The EPA program requires nurses to attend language and practical training programs in Japan. During practical training, they can work as nurse assistants (candidates) to understand the daily nursing process, laws, and Japanese medical system. Additionally, Japan has a history of recruiting foreign-educated nurses from other countries such as China, Korea, and Mongolia. Unlike recruiting under the EPA program, training is not required for these nurses [6].

Studies have shown that migrants face many challenges in integrating and adapting to the working environments of receiving countries because of communication barriers, skill underutilization, and discrimination, all of which can affect their integration process [7–9]. Among these challenges, workplace discrimination from colleagues, managers, and patients has been frequently reported among foreign-educated nurses [10]. Workplace discrimination refers to unfair terms and preferences regarding personal characteristics such as race, sex, religion, and social status that impair the ability of individuals [11]; it has a serious effect on foreign-educated nurses' physical and mental health, making them feel unaccepted and devalued in the organization and team, impacting patient safety and reducing their job satisfaction [9].

Job satisfaction is an indicator of hospital performance in managing multicultural nursing workplaces [12] and is defined as the degree to which a nurse's job fulfills their perceived needs [13]. Numerous factors contribute to nurses' job satisfaction, including age, sex, supervisor [14], job position, and hospital retirement plan [15]. The intention to leave is linked to nurses' turnover behavior, which harms hospital management by influencing the quality of care [14]. Foreign-educated nurses' intentions to leave include not only leaving the organization or profession but also returning to their countries of origin. The turnover of foreign-educated nurses has a negative impact on employers, especially short-term turnover, which can increase the cost of human resources in hospitals [16].

Research focused on factors related to foreign-educated nurses' job satisfaction or intention to leave has been conducted worldwide [17–20]. A nationwide study of 1951 foreign-educated nurses in Canada found that the ones who were young and had fewer years of residence and nursing practice had higher job satisfaction than their counterparts [19]. Kim et al. [18] investigated 165 Korean nurses in the United States (US) and reported that the more the nurses were satisfied with their organizational commitment and culture, the more satisfied they were with their job. Alreshidi et al. [17] found that foreign-educated nurses in Saudi Arabia who were male, with higher education, and with fewer years of nursing experience (1–4 years) had higher turnover intentions than their counterparts. Perceived quality of orientation predicted organizational- and unit-level turnover intentions among 201 Asian nurses working in the US [20]. However, no studies have investigated job satisfaction or intention to leave among foreign-educated nurses in Japan.

Although the relationship between native nurses' job satisfaction and the intention to leave has been widely investigated, to the best of our knowledge, only one study has investigated the association between foreign-educated nurses' job satisfaction and intention to leave [21]. Goh and Lopez [21] reported that foreign-educated nurses in Singapore who wanted to leave their jobs had significantly lower job satisfaction than those who did not. Furthermore, no study has explored the role of job satisfaction as a mediator in the associations between factors such as workplace discrimination, language ability, demographic characteristics, and intention to leave among foreign-educated nurses.

There has been no research conducted in Japan to explore the correlation between job satisfaction and intention to leave among foreign-educated nurses, nor to identify the factors directly and indirectly linked to job satisfaction or intention to leave. By filling these gaps, this study could provide insights needed to develop a program or strategy for the adaptation and integration of foreign-educated nurses into the workplace environment.

## 2. Framework and Purpose

### 2.1. Theoretical Framework

**Figure 1:**
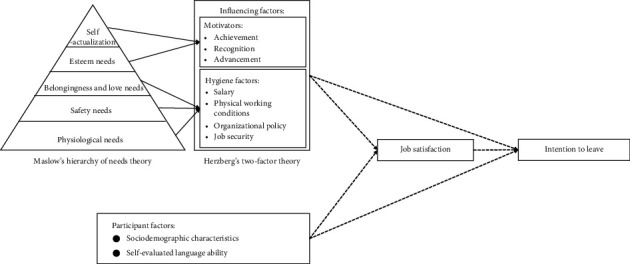
Theoretical framework.

Figure 1 shows this study's theoretical framework. The association between the influencing factors and the outcome variable (job satisfaction) was established based on Herzberg's two-factor theory [22]. Herzberg's two-factor theory, developed from Maslow's five-level hierarchy of needs theory, indicates that motivators and hygiene factors are two groups of elements that influence job satisfaction and dissatisfaction. The motivator directly corresponds to Maslow's highest level of need by asking “What do foreign-educated nurses want from their job?” This includes achievement, recognition, and advancement. Hygiene factors relate to Maslow's lowest needs level surrounding the job, including salary, physical working conditions, organizational policy and administration, and job security. Workplace discrimination was chosen as a hygiene factor because it is an important factor in protecting employees' job security [23]. Although the theory was constructed in 1959, it is still useful in many nursing studies today [24, 25].

The participant factors revealed foreign-educated nurses' characteristics based on previous studies, including sociodemographic characteristics and self-evaluated language ability. Self-evaluated language ability was chosen because previous research revealed that the language barrier was the most difficult for foreign-educated nurses to overcome in their new working environment [8]. Previous studies have empirically supported the association between job satisfaction and the intention to leave [14, 26]. Since the influencing or participant factors that affect foreign-educated nurses' job satisfaction and intention to leave may differ, the path relationship in Figure 1 is shown as a dotted line to demonstrate the exploratory nature of this study.

### 2.2. Purposes and Research Questions

This study aims to examine the relationship between job satisfaction and the intention to leave as well as identify the factors related to job satisfaction and the intention to leave among foreign-educated nurses in Japan.

We addressed the following research questions:

1. What is the level of job satisfaction and intention to leave among foreign-educated nurses in Japan?
2. What factors are related to their job satisfaction and intention to leave?
3. Is there a relationship between job satisfaction and intention to leave?

## 3. Materials and Methods

### 3.1. Study Design

A cross-sectional study was conducted in Japan between June and August 2022. It followed the Strengthening the Reporting of Observational Studies in Epidemiology (STROBE) guidelines for reporting observational studies.

### 3.2. Participants

This study recruited participants who met the following eligibility criteria: those who (1) were born and received basic nursing education outside of Japan; (2) had a Japanese registered nursing license; (3) were working as a nurse in Japan at the time of investigation; and (4) volunteered to participate in the study. The questionnaire was distributed to the participants by (1) e-mail including a survey link, through two organizations (one of the organizations actively trains foreign-educated nurses and recruits them; another is an organization for Chinese nurses in Japan), and (2) SNS messages (WeChat and Facebook) with a survey link that were sent through snowball sampling.

### 3.3. Sample Size and Power

This study's sample size was calculated using G*∗*Power. In total, 160 participants were needed for the correlation study with a power level of 0.90, a significance level of 5%, and an effect size (*r*) of 0.25. The selection of the effect size was based on a prior study which explored the association between foreign-educated nurses' job satisfaction and the intention to leave in Singapore [21].

### 3.4. Data Collection

Data were collected through a self-reported online Google Forms questionnaire. The questionnaire was available in both English and Japanese. To ensure accuracy, logical flow, readability, and ease of use, the questionnaire was piloted by members of our research team (including one nursing research expert and two fully experienced foreign-educated nurses). It took approximately 10–20 minutes to complete the questionnaire.

### 3.5. Variables and Measurement

The questionnaire comprised 8 sections with a total of 67 items. Items regarding influencing factors (hygiene factors and motivators) were chosen from a previous study that identified the factors related to nurses' job satisfaction according to Herzberg's two-factor theory [27] and studies that revealed the factors related to nurses' job satisfaction [15, 28]. Participant factors including sociodemographic characteristics and self-evaluated language ability were adapted from previous studies on foreign-educated nurses [18, 29].

#### 3.5.1. Hygiene Factors and Motivators

Hygiene factors included orientation for foreign-educated nurses (received or not), working shift (i.e., day shift, night shift, both, or others), yearly gross salary, employment contract (i.e., no fixed contract period, <3 years, ≥3 years but <5 years, ≥5 years, and do not know the period), and workplace discrimination. Workplace discrimination was measured using a one-item question asking participants whether they perceived themselves as having experienced discrimination in the workplace [10]. A 5-point scale was used to rate the discrimination level (1 = rarely or never to 5 = very often or continuously), with higher scores indicating higher discrimination.

Motivators included current position (i.e., staff nurse, head nurse, deputy director, and others), nursing specialist course (yes vs. no), and setting (i.e., hospital, institute, clinic, and others).

#### 3.5.2. Participant Factors

Sociodemographic characteristics included age, sex, marital status, education level, country of origin, country of obtaining basic (first-time) nursing education, years of residence and nursing practice in Japan, Japanese permanent residence (yes vs. no), living situation (living with someone or not), area of work (in Japan), and working experience in their country of origin (yes, >2 years; yes, <2 years; and no).

Self-evaluated language ability included language confidence and language satisfaction. Regarding language confidence, participants rated their perceptions of how confident they were in their current proficiency in Japanese language on a 4-point scale (4 = confident, 3 = somewhat confident, 2 = not very confident, and 1 = unconfident) [30], and regarding language satisfaction, the participants assessed their satisfaction with their current proficiency in Japanese language on a 5-point Likert scale (5 = very satisfied to 1 = very dissatisfied) [31].

#### 3.5.3. Job Satisfaction

The Japanese version of the Mueller–McCloskey Satisfaction Scale (MMSS) was used to measure job satisfaction [32]. The MMSS consists of 31 items from 8 dimensions: extrinsic rewards, scheduling, family/work balance, coworkers, interaction, professional opportunities, praise/recognition, and control/responsibility. Each item was measured on a 5-point Likert scale, and the total score ranged from 31 to 155 (a high score indicated a high job satisfaction level). The scale has been translated into Japanese, and its reliability and validity have been examined in Japan [33]. Cronbach's alpha of the MMSS scale is 0.89 in the original version and 0.90 in the Japanese version. The researchers obtained permission to use this scale from the creator and the Japanese translator. Cronbach's alpha for the scale in this study was 0.94, which also showed good reliability.

#### 3.5.4. Intention to Leave

Based on a previous study, the intention to leave was measured through three dimensions: current organization, nursing profession, and Japan [34]. Participants were asked to rate their intention to leave through the following questions: “Are you considering leaving (1) your current organization, (2) nursing profession, or (3) Japan in the coming year?” The responses were scored as 1 = very unlikely or rare, 2 = unlikely, 3 = highly likely, or 4 = very likely. The possible score ranged from 3 to 12, with lower scores representing a lower intention to leave. Cronbach's alpha for the scale in the current study was 0.74.

### 3.6. Data Analysis

Statistics like mean, median, and standard deviation were used to describe the study variables. The normality of the dependent variable (job satisfaction and intention to leave) was examined using the Shapiro–Wilk test and skewness and kurtosis distribution (±2). Job satisfaction and intention to leave were not normally distributed (*p* < 0.05) in the Shapiro–Wilk test, but the skewness and kurtosis values of job satisfaction (total and sub) were within 2 (the normal Q-Q plot was checked). Hence, job satisfaction was treated as an interval variable. Statistical analysis comprised two phases: the first phase was to identify the factors associated with job satisfaction or the intention to leave using SPSS, version 27 (IBM Corp.), and the second phase was to test the theoretical framework model using structural equation modeling (SEM) through IBM Amos version 28. The level of statistical significance was set at *p* < 0.05.

Bivariate and multiple regression analyses were conducted in the first phase. An independent *t*-test or analysis of variance was conducted to compare job satisfaction difference among nominal variables (e.g., sex, marital status, and nationality), whereas the Mann–Whitney *U* test or Kruskal–Wallis test was used to compare the differences in intention to leave among nominal variables. Furthermore, the Pearson correlation test was used to examine the association among job satisfaction, age, years of residence and practice in Japan, education level, nursing experience in the country of origin, turnover experience, workplace discrimination, language confidence, and language satisfaction, whereas Spearman's correlation coefficients were used to analyze the association between ordinal variables of intention to leave and the same variables. Depending on the number of analyses, Bonferroni correction was used for multiple comparisons (e.g., 0.05/3 = 0.017).

Variables with a *p* value <0.25 in the bivariate analysis were selected as candidates for forward multivariate regression to reduce potential bias and fulfill the regression model [35]. Linear regression was used to determine factors related to job satisfaction. Regarding the intention to leave, the 4-point Likert scale was collapsed into binary categories of intention to remain (very unlikely or rare and unlikely) and intention to leave (highly likely and very likely) to examine the factors related to the intention to leave through binary logistic regression. Moreover, the three domains were analyzed separately to better understand foreign-educated nurses' intentions to leave. Associations among the added variables were checked using bivariate analysis to adjust the model and avoid multicollinearity, as well as the variance inflation factor (VIF) (VIF < 5) in linear regression. In the bivariate and multivariate analyses, missing values were deleted for each pair.

Variables that were significant (*p* < 0.05) in the regression analysis (phrase one) were selected for phase two to verify the path and synthetic relationship among the theoretical framework models through SEM. According to Kline [36], the SEM case ratio should be >10 : 1. After removing missing data, 167 cases were available to assess the model with 10 parameters and an acceptable ratio of 17 : 1. The maximum likelihood method was used to estimate the covariance matrix, and the 2000 times bootstrap test was used to test the indirect effects. The fit indices used to evaluate the SEM model were chi-square (*χ*^2^, *p* > 0.05), root mean square error of approximation (RMSEA < 0.05), standardized root mean squared residual (SRMR < 0.10), and comparative fit index (CFI > 0.90) [36]. In the discussion, an online *t*-test calculator [37] was used to compare job satisfaction level and intention to leave with previous studies.

### 3.7. Ethical Consideration

The study was approved by the Institutional Review Board (IRB) of the Institute of Education of Tokyo Medical and Dental University (IRB number: C2021-013). The purpose of the study was explained to the participants at the beginning of the online questionnaire, and clicking the “Agree, approval to participate” button indicated that the participants understood the aim and consented to participate. Participants were informed that their participation was voluntary and that their responses would be kept anonymous.

To compensate the participants for their time and effort, an Amazon gift card (worth $4) was sent to those who provided their e-mail address after completing the questionnaire. E-mail addresses for sending the gift card were collected using a separate Google Form, and participants were informed about their right to provide their e-mail address or not.

## 4. Results

### 4.1. Participant Characteristics

In total, 206 foreign-educated nurses responded to the questionnaire. Data from 180 participants (effective response rate: 87.4%) were used for the analysis because 26 participants did not meet the inclusion criteria.  Table 1 shows the participants' demographic characteristics. Participants were Chinese (*n* = 178), Taiwanese (*n* = 1), and Indonesian (*n* = 1). Most participants were women (91%) and worked as staff nurses (98%) in Japan. The mean age of all participants was 29.6 ± 3.6 years, and the mean years of residence and nursing practice in Japan were 6.4 ± 3.3 and 4.5 ± 2.4 years, respectively. The average score of job satisfaction was 94.1 ± 17.6 (item mean: 3.0), with a wide range of 31.0 to 145.0, indicating a moderate satisfaction level. In terms of the intention to leave, the median of intention to leave the current organization, the nursing profession, and Japan was 2.0.

### 4.2. Factors Associated with Job Satisfaction

Table 1 shows that married nurses reported higher job satisfaction than single nurses did (*t* = −2.22, *p* = 0.028). Foreign-educated nurses who had received orientation from their current organization showed higher job satisfaction than those who had not (*t* = 2.10, *p* = 0.037). Night-shift nurses had the lowest job satisfaction scores (*f* = 6.45, *p* < 0.001).  Table 2 presents the results of the correlation analyses. Years of nursing practice in Japan (*r* = 0.21, *p* = 0.006), nursing experience in the country of origin (*r* = 0.17, *p* = 0.037), language confidence (*r* = 0.18, *p* = 0.015), and language satisfaction (*r* = 0.24, *p* = 0.027) were positively correlated with job satisfaction. Workplace discrimination was negatively correlated with job satisfaction (*r* = −0.44, *p* < 0.001).

Language confidence is strongly correlated with language satisfaction (*r* = 0.62, *p* < 0.001). Therefore, only language satisfaction was included in the final regression model because language confidence was not significant after controlling for other variables. The predictors of job satisfaction in the linear regression analysis (*R*^2^ = 0.373) are presented in Table 3. Workplace discrimination (*β* = −0.32, *p* < 0.001) was a negative predictor of job satisfaction, whereas satisfaction with Japanese (*β* = 0.17, *p* = 0.024) and receiving orientation (*β* = 0.16, *p* = 0.010) were positive predictors of job satisfaction. Compared to nurses who worked two shifts, only night-shift nurses had lower job satisfaction (*β* = −0.22, *p* = 0.001), but nurses who worked more flexible hours like short-time or part-time had higher job satisfaction (*β* = 0.15, *p* = 0.027).

### 4.3. Factors Associated with the Intention to Leave

Single nurses had a higher intention to leave compared to married nurses across three domains (current organization: *z* = −3.50, *p* < 0.001; nursing profession: *z* = −3.14, *p* = 0.002; Japan: *z* = −3.82, *p* < 0.001). Nurses with permanent residence in Japan were less likely to leave their current organization (*z* = −2.62, *p* = 0.009) or Japan (*z* = −2.73, *p* = 0.006) than those without it. Nurses who lived with their families were less likely to leave the nursing profession (*h* = 10.93, *p* = 0.012) or Japan (*h* = 13.46, *p* = 0.004) than those who lived alone or with friends. Nurses who received the specialist course in Japan reported a lower intention to leave their current organization (*z* = −2.08, *p* = 0.037) (Table 1). Younger age, fewer years of nursing practice and residence in Japan, and lower job satisfaction were related to a higher intention to leave across all three domains. Furthermore, workplace discrimination was positively associated with the intention to leave the current organization (*ρ* = 0.21, *p* < 0.01) (Table 4).

According to the Spearman correlation coefficients, age, years of residence, and nursing practice in Japan were highly correlated with each other (*ρ* > 0.70, *p* < 0.001). The final model only included years of nursing practice in Japan because it contributed to a higher variance in the model than the other two variables. Living with someone was excluded from the final model because it correlated with marital status (VIF > 5).  Table 5 shows the results of the binary logistic regression analysis. Fewer years of practice in Japan (odds ratio (OR) [95% confidence interval (CI)] = 0.45 [0.25–0.79], *p* = 0.006) and lower job satisfaction (OR [95% CI] = 0.96 [0.94–0.99], *p* = 0.006) predicted a higher intention to leave the current organization. Higher satisfaction with Japanese language (OR [95% CI] = 1.78 [1.18–2.68], *p* = 0.006) and single nurses (OR [95% CI] = 0.27 [0.09–0.81], *p* = 0.020) showed a higher intention to leave the nursing profession. Moreover, single nurses were more likely than married nurses to plan to leave Japan (OR [95% CI] = 0.11 [0.02–0.66], *p* = 0.016).

### 4.4. Final Model and Path Relationship among Variables

**Figure 2:**
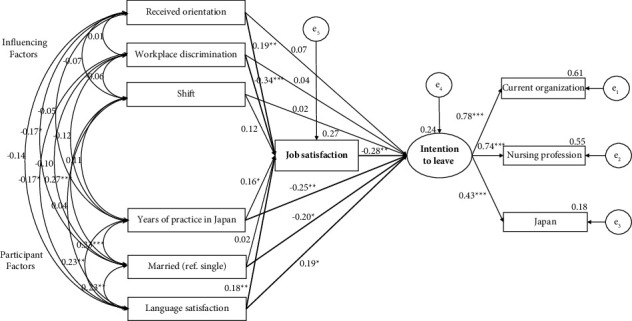
Final model and path relationship (*n* = 167).

Regarding the latent factor of the intention to leave, the standardized factor loadings were statistically significant, with magnitudes ranging from 0.78 (current organization) to 0.43 (Japan) (Figure 2). The final SEM model indicated a satisfactory model fit: chi-square (*χ*^2^) = 17.462, degree of freedom (DF) = 14, *p* = 0.232; RMSEA = 0.037; SRMR = 0.0313; and CFI = 0.984. Received orientation (*β* = 0.19, *p* = 0.005), less workplace discrimination (*β* = −0.34, *p* < 0.001), and higher language satisfaction (*β* = 0.18, *p* = 0.009) were directly related to higher job satisfaction. Fewer years of nursing practice in Japan (*β* = −0.25, *p* = 0.006), single status (*β* = −0.20, *p* = 0.027), and higher satisfaction with Japanese language (*β* = 0.19, *p* = 0.029) were directly related to a higher intention to leave. The model estimated 27.2% and 24.3% variances in job satisfaction and intention to leave, respectively.

Table 6 reveals the indirect relationship as follows: through job satisfaction, received orientation (*β* = −0.05, *p* = 0.018), years of practice in Japan (*β* = −0.05, *p* = 0.017), and language satisfaction (*β* = −0.05, *p* = 0.031) had a small negative indirect effect on the intention to leave. Workplace discrimination had a positive indirect effect on intention to leave (*β* = 0.09, *p* = 0.005).

## 5. Discussion

Using Herzberg's two-factor theory as our guiding framework, this is the first study to use SEM, a more rigorous analytic technique, to explore the relationship between foreign-educated nurses' job satisfaction and the intention to leave, as well as to identify the factors associated with those two variables. The results confirmed that higher workplace discrimination, lower Japanese language satisfaction, and not receiving orientation were predictors of foreign-educated nurses' lower job satisfaction. Lower job satisfaction, fewer years of nursing practice in Japan, single status, and higher language satisfaction predicted higher intention to leave. Moreover, workplace discrimination and receiving orientation had significant indirect effects on the intention to leave, with job satisfaction acting as a mediator.

This study revealed that foreign-educated nurses had a moderate level of job satisfaction in Japan, which was significantly higher than that reported in a previous study of 1241 Japanese hospital nurses (3.0 vs. 2.8, *t* = 3.3, *p* = 0.001) [37, 38]. This may be explained by the fact that the availability of stable jobs fulfills foreign-educated nurses' migrant motivation, as they can have better economic benefits and social status than those in their countries of origin [8]. The job satisfaction level in this study was lower than that in the study on 602 foreign-educated nurses (3.0 vs. 3.3, *t* = 17.9, *p* < 0.001) in Saudi Arabia [29, 37], but it was similar to that on 210 foreign-educated nurses (3.0 vs. 2.9, *t* = 1.3 *p* = 0.185) in Ireland [37, 39]. Healthcare systems and workloads vary across countries, which may cause foreign-educated nurses in various countries to fulfill their jobs differently. This also highlights the necessity of investigating foreign-educated nurses' job satisfaction in different receiving countries.

The present study's results showed that foreign-educated nurses had moderate to high intentions of leaving their current organization (2.4 vs. 1.8, *t* = 8.2, *p* < 0.001) and nursing profession (1.9 vs. 1.5, *t* = 6.9, *p* < 0.001), which was higher than those shown in a national study of 1261 Japanese nurses [37, 40]. This is probably due to the high turnover rate of foreign workers in Japan. Japanese national reports indicated that foreign turnover rates are twice as high as those of Japanese workers [41]. The high intention to leave among foreign-educated nurses was considered to be due to the inability to form human relations and adapt to the corporate culture in Japan [42].

In this study, workplace discrimination was the main predictor of job dissatisfaction. This is consistent with the results of a Canadian study of 1952 foreign-educated nurses that found that those who experienced discrimination were less satisfied with their jobs [19]. Discriminatory treatment in the workplace is an additional stressor that reduces foreign-educated nurses' self-esteem, confidence, and well-being and causes negative work attitudes [9]. Foreign-educated nurses who received orientation from their current organization showed higher job satisfaction than those who did not. A similar finding was reported by a US study, which showed that Asian nurses who received orientation with people of similar cultural backgrounds were more satisfied with their practice environment and had more organizational commitment than those who did not [43].

The positive correlation between language satisfaction and job satisfaction was supported by previous qualitative studies that identified language barriers as a crucial problem influencing foreign-educated nurses' job satisfaction [44]. Foreign-educated nurses reported that they face challenges in communicating with patients during nursing care and sought improvement in their language abilities to provide appropriate care and responses to patients [44, 45]. However, nurses with higher language satisfaction were found to have a greater intention to leave their nursing role. The lack of career development opportunities may explain this finding. Foreign-educated nurses have fewer opportunities to apply for management positions or professional development than native nurses [46]. Nurses with good language proficiency likely think there are no opportunities for career development in nursing and plan to leave their nursing roles.

Through SEM, it was found that years of nursing practice have a positive correlation with job satisfaction and a negative correlation with the intention to leave. Time is reported to be particularly important for foreign-educated nurses' adaptation and integration processes [28]. During the early stages of migration, foreign-educated nurses leave their countries of origin and struggle to adapt to the new work environment and culture, which might result in low resilience and job dissatisfaction [45]. High intention to leave during the initial years of nursing practice may also be explained by the failure to adapt. Hence, specific support for foreign-educated nurses to successfully adapt to their new work environment during their early immigrant years is required to enhance their job satisfaction and retention.

In this study, married nurses showed a lower intention to leave than single nurses, similar to a previous study on Korean nurses in the US [18]. Compared to married nurses, single nurses experience more life changes that require work adjustment, such as moving to a place they are interested in, getting married, and seeking jobs with work-life balance [47]. Moreover, married nurses with children and financial burdens (e.g., house loans) likely find it difficult to make turnover decisions and want to remain stable [18]. Single foreign-educated nurses may feel isolated and seek family support, which prompts them to return to their countries of origin.

Through SEM, the intention to leave was regarded as a latent variable consisting of three domains that each loaded >0.40, which is acceptable [36]. Foreign-educated nurses with lower job satisfaction showed a higher intention to leave, and job satisfaction mediated the relationship between receiving orientation, workplace discrimination, years of nursing practice in Japan, language satisfaction, and intention to leave. This result is consistent with a previous study among nurses in general [14], indicating that job satisfaction plays a key role in preventing nurses' turnover intentions. However, according to the results of a logistic regression analysis, lower job satisfaction predicted only the intention to leave the current organization and was not statistically significant concerning the intention to leave the nursing profession or Japan. This finding indicates that other factors specifically related to foreign-educated nurses' intentions to leave the nursing profession or Japan have not been measured. Further studies should fill this scholarly gap and contribute to a deeper understanding of turnover among foreign-educated nurses.

### 5.1. Implication for Nursing Management

Nurse managers and policymakers should develop strategies to prevent workplace discrimination and provide career advancement opportunities to ensure a positive and equal work environment for foreign-educated nurses. Counseling services should be available for those who face challenges caused by cultural differences or workplace discrimination. Moreover, educational programs such as short-term language training to familiarize with Japanese nursing documents, long-term language training to improve communication skills for cross-cultural nurse-patient interaction, and continuing education programs to learn about Japanese nursing culture might help foreign-educated nurses better understand their roles in the new country.

Since most of the study's participants were from China, there are cultural (e.g., Chinese people prefer warm water while Japanese people prefer ice water to drink) and nursing care differences (e.g., bedside daily care in China is mainly provided by patients' families or care assistants while Japanese nursing duties include whole patient daily care, such as feeding and bathing) [48]. Adequate job orientation programs (e.g., how to provide patient daily care) from employers could assist Chinese-educated nurses in gaining knowledge and skills to adapt to new work environments, enhancing their job satisfaction and reduce turnover in Japan. Providing information comparing Japanese-Chinese nursing and culture to Japanese employers and nurse managers would also help them better understand Chinese-educated nurses, eliminate stereotype-based discrimination (e.g., Chinese nurses cannot provide patients' daily care), and establish adequate support strategies.

Furthermore, managers should develop specific strategies to monitor and support nurses who are at a higher risk of turnover and dissatisfaction, such as those who are single or have less nursing experience. The possible strategies considered were (1) supporting experienced nurses with career development or educational resources; (2) monitoring their job satisfaction and turnover intentions over time; and (3) developing cultural or nursing condition exchange activities with native nurses to support their adaptation.

### 5.2. Limitations and Further Research Implications

This study has several limitations. The first is the use of a cross-sectional design, which limits the ability to establish causal relationships. Moreover, the mediation test implies that the longitudinal model is more appropriate than cross-sectional data [49]. Thus, further research using a longitudinal study design is recommended to clarify the mediating role of job satisfaction among foreign-educated nurses. Second, most participants (99%) were Chinese; hence, this study's results cannot be generalized to nurses of other nationalities. Further studies should collect data from foreign-educated nurses of diverse nationalities to address this issue. Third, night-shift nurses showed the lowest job satisfaction; however, data from only two nurses are not sufficient to draw a clear relationship between shift and job satisfaction. Further studies with larger sample sizes are warranted. Finally, nurses with low job satisfaction and high intentions to leave may have already left and were not included in this survey. It would be necessary to survey nurses who have already left, especially those who have left the nursing profession and Japan.

## 6. Conclusion

This study contributes to the understanding of foreign-educated nurses' job satisfaction and intention to leave. Overall, foreign-educated nurses were moderately satisfied with their jobs in Japan. Higher job satisfaction was shown by foreign-educated nurses who perceived less workplace discrimination, received orientation, and perceived higher satisfaction with Japanese language. They were more likely to leave if they had lower job satisfaction, higher language satisfaction, fewer years of nursing practice in Japan, and were single. Providing a positive work environment by reducing workplace discrimination and providing adequate orientation as well as language support training and counseling services might enhance foreign-educated nurses' job satisfaction and retention.

## Figures and Tables

**Table 1:** Participant characteristics (*n* = 180).

Variables	*n*	Job satisfaction		Intention to leave (current organization)		Intention to leave (nursing profession)^#^		Intention to leave (Japan)	
		Mean (SD)	*t*/*f*	Median [25−75^th^]	*z*/*h*	Median [25−75^th^]	*z*/*h*	Median [25−75^th^]	*z*/*h*
Gender			0.45^a^		−0.14^c^		−0.68^c^		−0.13^c^
Women	164	94.4 (15.8)		2.0 [2.0–3.0]		2.0 [1.0–3.0]		2.0 [1.0–2.0]	
Men	16	90.9 (30.2)		2.0 [1.0–3.0]		2.0 [1.0–3.0]		2.0 [1.0–2.0]	
Marital status			−2.22^a^^*∗*^		−3.50^c^^*∗∗∗*^		−3.14^c^^*∗∗*^		−3.82^c^^*∗∗∗*^
Single	115	91.9 (18.7)		3.0 [2.0– 3.0]		2.0 [1.0–3.0]		1.0 [1.0–2.0]	
Married	65	97.9 (14.8)		2.0 [1.0–3.0]		1.0 [1.0–2.0]		1.0 [1.0–2.0]	
Japanese permanent residence			−1.72^a^		−2.62^c^^*∗∗*^		−0.83^c^		−2.73^c^^*∗∗*^
Yes	17	101.0 (11.8)		2.0 [1.0–2.0]		1.0 [1.0–2.5]		1.0 [1.0–1.5]	
No	163	93.3 (17.9)		2.0 [2.0–3.0]		2.0 [1.0–3.0]		2.0 [1.0–1.0]	
Living with somebody			2.04^b^		10.93^d^^*∗*^		6.88^d^		13.46^d^^*∗∗*^
Living alone	95	91.5 (18.7)		2.0 [2.0–3.0]		2.0 [1.0–3.0]		2.0 [1.0–2.0]	
Living with partner only	34	93.9 (15.7)		2.5 [2.0–3.0]		1.5 [1.0–2.0]		2.0 [1.0–2.0]	
Living with family member (partner, child, and/or parents)	43	98.7 (14.8)		2.0 [1.0–2.25]		2.0 [1.0–2.0]		1.0 [1.0–2.0]	
Living with friends	8	100.0 (21.8)		3.0 [1.25–3.75]		2.0 [1.0–3.0]		1.5 [1.0–2.0]	
Area of work			0.71^b^		0.30^d^		2.02^d^		0.35^d^
Kanto	123	93.1 (17.2)		2.0 [2.0–3.0]		2.0 [1.0–3.0]		2.0 [1.0–2.0]	
Kinki	39	96.9 (16.0)		2.0 [1.5–3.0]		1.0 [1.0–2.0]		1.0 [1.0–2.0]	
Others	18	94.4 (17.5)		2.0 [1.25–3.75]		2.0 [1.0–2.75]		1.0 [1.0–2.0]	
Setting			0.36^b^		0.72^d^		2.09^d^		0.24^d^
Hospital	151	94.3 (17.6)		2.0 [1.5–3.0]		2.0 [1.0–3.0]		2.0 [1.0–2.0]	
Care institute	16	91.1 (16.2)		2.0 [2.0–3.0]		2.0 [2.0–3.0]		2.0 [1.0–2.0]	
Clinic	10	97.3 (18.6)		2.5 [2.0–3.0]		2.0 [1.0–3.0]		1.5 [1.0–2.25]	
Others	3	88.7 (27.7)		Only three participants in this group					
Orientation			2.10^a^^*∗*^		−0.24^c^		−0.82^c^		−0.33^c^
Yes	60	97.9 (16.3)		2.0 [2.0–3.0]		2.0 [1.0–3.0]		2.0 [1.0–2.0]	
No	120	92.1 (17.9)		2.0 [2.0–3.0]		2.0 [1.0–2.0]		2.0 [1.0–2.0]	
Annual income			0.10^b^		6.85^d^		4.27^d^		2.86^d^
<3 million yen	19	94.9 (17.6)		3.0 [1.5–4.0]		2.0 [1.0–3.0]		2.0 [1.0–2.0]	
3 million to <5 million yen	118	93.8 (17.0)		2.0 [2.0–3.0]		2.0 [1.0–3.0]		2.0 [1.0–2.0]	
≥5 million yen	37	95.0 (20.3)		2.0 [1.75–3.0]		2.0 [1.0–2.0]		2.0 [1.0–2.0]	
Prefer not to answer	6	91.5 (15.0)		1.0 [1.0–2.25]		1.0 [1.0–2.25]		2.0 [1.0–2.0]	
Specialist courses in Japan			0.95^a^		−2.08^c^^*∗*^		−0.64^c^		−1.96^c^
Yes	10	99.2 (12.4)		2.0 [1.0–3.0]		1.5 [1.0–2.25]		1.0 [1.0–2.0]	
No	170	93.7 (17.8)		2.0 [2.0–3.0]		2.0 [1.0–3.0]		2.0 [1.0–2.0]	
Employment contract			1.83^b^		5.14^d^		6.45^d^		4.63^d^
<3 years	31	88.5 (17.8)		2.0 [2.0–3.0]		2.0 [1.0–3.0]		2.0 [1.0–2.0]	
≥3 years	30	94.7 (18.2)		3.0 [1.5–3.5]		2.0 [1.0–3.0]		2.0 [1.0–2.0]	
No fixed contract period	100	96.6 (15.4)		2.0 [2.0–3.0]		2.0 [1.0–2.0]		2.0 [1.0–2.0]	
Do not know if there is a fixed contract period	19	90.6 (23.9)		2.0 [1.0–3.0]		2.0 [1.0–3.0]		2.0 [1.0–2.0]	
Shift			6.45^b^^*∗∗∗*^		8.69^d^		2.01^d^		4.52^d^
Two shifts	124	92.9 (16.3)		2.0 [2.0–3.0]		2.0 [1.0–3.0]		2.0 [1.0–2.0]	
Three shifts	10	98.4 (10.7)		3.0 [1.0–3.0]		2.0 [1.0–3.0]		2.0 [2.0–2.25]	
Day shift only	42	97.8 (18.2)		2.0 [1.0–3.0]		2.0 [1.0–2.5]		1.0 [1.0–2.0]	
Night shift only	2	44.0 (18.4)		Only two participants for each group					
Short-time or part-time	2	118.0 (19.8)							

^#^Missing data with 8 participants (<10% of the total sample), pairwise in the analysis. ^a^Student's *t*-test; ^b^one-way analysis of variance with Bonferroni correction; ^c^Mann–Whitney *U* test; ^d^Kruskal–Wallis test with Bonferroni correction. ^*∗*^*p* < 0.05; ^*∗∗*^*p* < 0.01; ^*∗∗∗*^*p* < 0.001. SD: standard deviation.

**Table 2:** Results of Pearson correlation test for factors associated with job satisfaction.

Variables	1	2	3	4	5	6	7	8	9
(1) Job satisfaction									
(2) Age	0.113								
(3) Years of residence in Japan^#^	0.076	0.741^*∗∗∗*^							
(4) Years of nursing practice in Japan^#^	0.207^*∗∗*^	0.681^*∗∗∗*^	0.766^*∗∗∗*^						
(5) Education level	0.082	0.027	−0.001	−0.023					
(6) Nursing experience in country of origin	0.165^*∗*^	0.078	−0.045	−0.053	−0.007				
(7) Turnover experience	−0.021	0.325^*∗∗*^	0.299^*∗∗*^	0.335	−0.05	−0.077			
(8) Workplace discrimination	−0.440^*∗∗∗*^	−0.080	−0.075	−0.128	0.063	−0.094	0.121		
(9) Language confidence	0.182^*∗*^	0.111	0.239^*∗∗*^	0.158^*∗*^	0.119	0.041	0.127	−0.170^*∗*^	
(10) Language satisfaction	0.235^*∗∗*^	0.220^*∗∗*^	0.309^*∗∗*^	0.248^*∗∗∗*^	0.069	0.024	0.188^*∗*^	0.195^*∗*^	0.624^*∗∗∗*^

^#^Missing data with 5 participants (<10% of the total sample), pairwise in the analysis. ^*∗*^*p* < 0.05; ^*∗∗*^*p* < 0.01; ^*∗∗∗*^*p* < 0.001.

**Table 3:** Predictors of job satisfaction according to linear regression analysis.

Variables	*B*	*β*	*p*	*R * ^2^	VIF
Workplace discrimination	−6.24	−0.32	<0.001	0.189	1.23
Received orientation (ref. did not receive)	6.53	0.17	0.010	0.024	1.13
Language satisfaction	2.54	0.16	0.024	0.034	1.24
Nursing experience in country of origin	2.48	0.09	0.181	0.010	1.17
Years of nursing practice in Japan	3.11	0.14	0.071	0.018	1.38
Having Japanese permanent residence (ref. not having)	1.22	0.02	0.803	0.006	1.57
Married (ref. single)	2.65	0.07	0.598	0.001	4.68
Living with somebody (ref. living alone)				0.014	
Living with partner only	−2.28	−0.05	0.597		2.32
Living with family (partner, child, and/or parents)	−1.86	−0.05	0.748		4.87
Living with friends	10.19	0.11	0.085		1.07
Shift (ref. two shifts)				0.076	
Three shifts	3.38	0.04	0.506		1.10
Day shifts only	3.49	0.08	0.216		1.15
Night shifts only	−36.76	−0.22	0.001		1.14
Other (i.e., part-time)	24.13	0.15	0.027		1.06
Employment contract (ref. no fixed contract)				0.002	
<3 years	−1.64	−0.04	0.628		1.30
≥3 years	0.20	0.00	0.952		1.19
Do not know if there is a fixed contract period	−2.00	−0.03	0.622		1.15
Constant	79.28				

Linear regression analysis (enter): *R*^2^ = 0.373; *F* = 5.499; Durbin–Watson value = 2.12; ref.: reference; VIF: variance inflation factor.

**Table 4:** Results of Spearman correlation test for factors associated with the intention to leave the current organization/nursing profession/Japan.

Variables	1	2	3	4	5	6	7	8	9	10	11	12
(1) Intention to leave current organization												
(2) Intention to leave nursing profession	0.549^*∗∗∗*^											
(3) Intention to leave Japan	0.369^*∗∗∗*^	0.519^*∗∗∗*^										
(4) Age	−0.237^*∗∗*^	−0.213^*∗∗*^	−0.190^*∗*^									
(5) Years of residence in Japan^#^	−0.211^*∗∗*^	−0.212^*∗∗*^	−0.198^*∗∗*^	0.775^*∗∗∗*^								
(6) Years of nursing practice in Japan^#^	−0.344^*∗∗∗*^	−0.270^*∗∗∗*^	−0.239^*∗∗*^	0.697^*∗∗∗*^	0.776^*∗∗∗*^							
(7) Education level	0.008	−0.034	0.046	0.009	−0.021	−0.016						
(8) Nursing experience in country of origin	−0.028	0.029	0.059	0.05	−0.068	−0.078	−0.009					
(9) Turnover experience	−0.085	−0.047	−0.105	0.314^*∗∗∗*^	0.299^*∗∗*^	0.347^*∗∗∗*^	−0.052	−0.093				
(10) Job satisfaction	−0.369^*∗∗∗*^	−0.243^*∗∗*^	−0.197^*∗∗*^	0.084	0.066	0.200^*∗∗*^	0.076	0.161^*∗*^	−0.027			
(11) Workplace discrimination	0.209^*∗∗*^	0.123	0.119	−0.088	−0.073	−0.114	0.056	−0.09	0.101	−0.444^*∗∗∗*^		
(12) Language confidence	−0.049	0.062	−0.083	0.096	0.219^*∗∗*^	0.163^*∗*^	0.109	0.034	0.132	0.143	−0.166^*∗*^	
(13) Language satisfaction	−0.098	0.063	−0.115	0.211^*∗∗*^	0.300^*∗∗∗*^	0.254^*∗∗∗*^	0.121	−0.01	0.191^*∗*^	0.247^*∗*^	−0.185^*∗*^	0.610^*∗∗∗*^

^#^Missing data with 5 participants (<10% of the total sample), pairwise in the analysis. ^*∗*^*p* < 0.05; ^*∗∗*^*p* < 0.01; ^*∗∗∗*^*p* < 0.001.

**Table 5:** Predictors of the intention to leave according to binary logistic regression analysis.

Variables	Current organization			Nursing profession			Japan		
	OR [95% CI]	*p*	VIF	OR [95% CI]	*p*	VIF	OR [95% CI]	*p*	VIF
Job satisfaction	0.96 [0.94–0.99]	0.006	1.49	0.98 [0.95–1.01]	0.110	1.36	1.01 [0.97–1.04]	0.764	1.49
Years of nursing practice in Japan	0.45 [0.25–0.79]	0.006	1.59	0.54 [0.27–1.07]	0.076	1.62	0.88 [0.39–1.95]	0.744	1.59
Language satisfaction	1.16 [0.81–1.67]	0.404	1.30	1.78 [1.18–2.68]	0.006	1.28	1.16 [0.73–1.84]	0.543	1.30
Married (ref. single)	0.71 [0.30–2.49]	0.414	1.20	0.27 [0.09–0.81]	0.020	1.18	0.11 [0.02–0.66]	0.016	1.20
Did not receive nursing specialty course in Japan (ref. received)	0.11 [0.10–1.16]	0.064	1.05	1.21 [0.19–7.66]	0.843	1.05	0.00 [0.00–0.00]	0.999	1.05
No Japanese permanent residence (ref. yes)	0.69 [0.14–3.49]	0.655	1.47	3.96 [0.75–20.87]	0.104	1.51	2.26 [0.17–30.74]	0.541	1.47
Workplace discrimination	1.17 [0.74–1.85]	0.499	1.35	1.14 [0.68–1.91]	0.611	1.27	1.60 [0.89–2.91]	0.119	1.35
Annual income (ref. <3 million)									
3 million to <5 million	0.56 [0.15–2.17]	0.403	3.14	2.81 [0.70–11.28]	0.144	3.06	1.65 [0.30–9.10]	0.564	3.14
≥5 million	0.98 [0.19–5.02]	0.980	3.36	1.24 [0.20–7.77]	0.815	3.32	1.99 [0.22–17.86]	0.538	3.35
Prefer not to answer	0.09 [0.07–1.32]	0.080	1.42	0.79 [0.05–12.16]	0.865	1.44	0.00 [0.00–0.00]	0.999	1.42
Shift (ref. two shifts)									
Three shifts	4.18 [0.78–22.29]	0.094	1.40	2.08 [0.46–9.35]	0.339	1.41	1.91 [0.31–11.92]	0.490	1.40
Day shifts only	0.80 [0.33–1.95]	0.626	1.11	1.33 [0.49–3.61]	0.580	1.11	1.45 [0.43–4.91]	0.550	1.11
Night shifts only	255164759 [0–0]	0.999	1.21	—	—	—	0.00 [0.00–0.00]	0.999	1.21
Others	2.35 [0.09–64.92]	0.614	1.26	0.00 [0.00–0.00]	1.00	1.22	0.00 [0.00–0.00]	0.999	1.26
Employment contract (ref. <3 years)									
≥3 years	2.16 [0.61–7.60]	0.278	1.26	0.88 [0.25–3.13]	0.838	1.26	1.38 [0.35–5.38]	0.643	1.26
No fixed contract period	1.28 [0.47–3.48]	0.665	1.25	0.67 [0.22–1.99]	0.470	1.24	0.65 [0.18–2.28]	0.498	1.25
Do not know if there is a fixed contract period	1.07 [0.23–5.07]	0.976	1.17	1.33 [0.30–5.81]	0.709	1.13	1.01 [0.14–7.40]	0.990	1.17
Hosmer and Lemeshow test (*χ*^2^, *p*)	4.580	0.801		9.390	0.310		5.561	0.696	
Nagelkerke *R*^2^	0.325			0.255			0.224		

Logistic regression analysis (enter) with outcome: total score of intention to leave current organization >2 (*n* = 83), ≤2 (*n* = 97); total score of intention to leave nursing profession >2 (*n* = 45), ≤2 (*n* = 127); total score of intention to leave Japan >2 (*n* = 25), ≤2 (*n* = 155). OR: odds ratio; CI: confidence interval; SE: standardized error; ref.: reference; VIF: variance inflation factor.

**Table 6:** Indirect effects of the structural equation modeling result (*n* = 167).

Structural path	*β*	*p*
Received orientation ⟶ job satisfaction ⟶ intention to leave	−0.053	0.018
Workplace discrimination ⟶ job satisfaction ⟶ intention to leave	0.093	0.005
Shift ⟶ job satisfaction ⟶ intention to leave	−0.033	0.155
Years of nursing practice in Japan ⟶ job satisfaction ⟶ intention to leave	−0.045	0.017
Married (ref. single) ⟶ job satisfaction ⟶ intention to leave	−0.007	0.741
Language satisfaction ⟶ job satisfaction ⟶ intention to leave	−0.050	0.031

## Data Availability

The data used to support the findings of this study are available from the corresponding author upon request.

## Supplementary Materials

Supplementary Materials The supplementary material is the result of Strengthening the Reporting of Observational Studies in Epidemiology (STROBE), which was used as guidance for this study.
